# Heterosubtypic Protection against Pathogenic Human and Avian Influenza Viruses via *In Vivo* Electroporation of Synthetic Consensus DNA Antigens

**DOI:** 10.1371/journal.pone.0002517

**Published:** 2008-06-25

**Authors:** Dominick J. Laddy, Jian Yan, Michele Kutzler, Darwyn Kobasa, Gary P. Kobinger, Amir S. Khan, Jack Greenhouse, Niranjan Y. Sardesai, Ruxandra Draghia-Akli, David B. Weiner

**Affiliations:** 1 Department of Pathology & Laboratory Medicine, University of Pennsylvania School of Medicine, Philadelphia, Pennsylvania, United States of America; 2 Respiratory Viruses, National Microbiology Laboratory, Public Health Agency of Canada, Winnipeg, Manitoba, Canada; 3 Special Pathogens, National Microbiology Laboratory, Public Health Agency of Canada, Winnipeg, Manitoba, Canada; 4 Department of Medical Microbiology, University of Manitoba, Winnipeg, Manitoba, Canada; 5 VGX Pharmaceuticals, Blue Bell, Pennsylvania, United States of America; 6 Bioqual, Inc., Rockville, Maryland, United States of America; University of California San Francisco, United States of America

## Abstract

**Background:**

The persistent evolution of highly pathogenic avian influenza (HPAI) highlights the need for novel vaccination techniques that can quickly and effectively respond to emerging viral threats. We evaluated the use of optimized consensus influenza antigens to provide broad protection against divergent strains of H5N1 influenza in three animal models of mice, ferrets, and non-human primates. We also evaluated the use of *in vivo* electroporation to deliver these vaccines to overcome the immunogenicity barrier encountered in larger animal models of vaccination.

**Methods and Findings:**

Mice, ferrets and non-human primates were immunized with consensus plasmids expressing H5 hemagglutinin (pH5HA), N1 neuraminidase (pN1NA), and nucleoprotein antigen (pNP). Dramatic IFN-γ-based cellular immune responses to both H5 and NP, largely dependent upon CD8+ T cells were seen in mice. Hemaggutination inhibition titers classically associated with protection (>1:40) were seen in all species. Responses in both ferrets and macaques demonstrate the ability of synthetic consensus antigens to induce antibodies capable of inhibiting divergent strains of the H5N1 subtype, and studies in the mouse and ferret demonstrate the ability of synthetic consensus vaccines to induce protection even in the absence of such neutralizing antibodies. After challenge, protection from morbidity and mortality was seen in mice and ferrets, with significant reductions in viral shedding and disease progression seen in vaccinated animals.

**Conclusions:**

By combining several consensus influenza antigens with *in vivo* electroporation, we demonstrate that these antigens induce both protective cellular and humoral immune responses in mice, ferrets and non-human primates. We also demonstrate the ability of these antigens to protect from both morbidity and mortality in a ferret model of HPAI, in both the presence and absence of neutralizing antibody, which will be critical in responding to the antigenic drift that will likely occur before these viruses cross the species barrier to humans.

## Introduction

Efforts to develop vaccines against highly pathogenic avian influenza (HPAI) highlight several challenges facing the vaccine development community. Predicting which strains of seasonal influenza to include in the annual vaccine is a difficult task, and has on multiple occasions led to the development of an ineffective or partially protective vaccine. This past year is a good example, with influenza vaccine coverage approximating a mere 30%. This prediction is made more difficult with H5N1 HPAI, whose evolution and migration have been shown to be more complex than was initially appreciated [1], [2]. The timeline for designing and producing conventional vaccines against an unpredicted emerging virus would preclude their development during an emerging epidemic [3]. In addition, humans have no pre-existing immunity to H5 viruses upon which to build, which may have contributed to the initial difficulty seen in inducing seroconversion to H5-based subunit and killed virus vaccines [4], [5], [6].

An ideal vaccine platform would include technologies that can be quickly and easily scaled up for mass production, in addition to a delivery mechanism that can quickly induce seroconversion against novel antigens. The induction of potent cross-reactive cellular responses, a challenge facing many vaccine platforms, could also prove very useful in augmenting absent or incomplete antibody neutralization.

Conceptually, DNA vaccines have many of these attributes. Their progress to the clinic, however, has been slowed by difficulties in reproducing the potent immune responses seen in small animals to other models of vaccination. In order to address the technical hurdles associated with limited vaccine immunogenicity, we have combined several highly optimized DNA vaccine constructs with constant-current *in vivo* electroporation (IVE) and analyzed immunogenicity in mouse, ferret, and primate models of vaccination. Electroporation has classically been used *in vitro* to enhance the delivery of plasmid to cells in culture. Recent studies, however, have shown its promise in enhancing the delivery and expression of plasmid DNA *in vivo*, leading to the generation of more potent immune responses [7], [8], [9].

In addition, we asked several important questions regarding vaccine-induced correlates of immunity to pathogenic influenza. These include the ability of cell-mediated immunity to protect against HPAI in the presence of the severe cytokine dysregulation associated with H5N1 influenza–a question thus far asked only in murine challenge models [10], [11], [12] and extended here to ferrets.

In order to address these questions, we have developed several consensus influenza antigens, several of which have been previously described [13]. These include an H5 hemagglutinin construct (pH5H1), whose component sequences include 16 predominantly clade 1 H5N1 sequences that have infected and proven fatal in humans and a consensus N1 neuraminidase construct (pN1NA), generated from over 40 influenza A sequences. We also present a construct based on consensus influenza A nucleoprotein (pNP), which has not been previously described.

## Materials and Methods

### DNA Vaccines

pH5HA and pN1NA have been previously described [13]. pNP was designed and constructed in a similar manner. Briefly, influenza A matrix 2 and nucleoprotein sequences were downloaded from the Los Alamos National Laboratory Influenza Sequence Database. Sequences were chosen from geographically diverse locations. MegAlign (DNASTAR, Madison, WI) was used to align the sequences and generate a consensus sequence. The consensus ectodomain of the matrix 2 antigen was fused to the full-length consensus nucleoprotein, and the construct optimized for expression, including codon and RNA optimization (GeneArt, Regensburg, Germany). The homology between these constructs and the original sequences is shown in Table 1. The construct elicits no antibody responses as determined by ELISA and HI. Each of the constructs was subcloned into the pVax expression vector. For the mouse studies, endotoxin-free DNA preparations were made using Qiagen Giga prep columns (Valencia, CA) [14]. For the ferret and non-human primate studies, DNA preparations were made at VGX Pharmaceuticals, Inc. (The Woodlands, TX) as previously described [7], and formulated at 10 mg/mL in water plus 1% w/w poly-L-glutamate sodium salt.

**Table 1:** Hemagglutination Inhibition of H5N1 Influenza Viruses.

	Clade 1		Clade 2.1
	2^nd^ Immunization	3^rd^ Immunization	3^rd^ Immunization
pNP	<20	<20	<20
pComb	170 (40–320)	600 (160–1280)	210 (40–320)
pVax	<20	<20	<20

Hemagglutination inhibition titers in ferrets after the second and third immunization against a clade 1 (A/Vietnam/1203/04) and clade 2.1 (A/Indonesia/05/2005) virus. Values shown include the mean and range of values.

#### Mouse Studies

The quadriceps muscles of 6–8 week old female BALB/C or C57BL/6 mice (Jackson Laboratory) were injected 3 times, two weeks apart, with 25 µg of plasmid-encoded antigen and electroporated as previously described [15]. Briefly, square-wave pulses were delivered through a triangular 3-electrode array consisting of 26-gauge solid stainless steel electrodes. Two constant-current pulses of 0.1 Amps were delivered for 52 msec/pulse separated by a 1 sec delay using the CELLECTRA® adaptive constant current device (VGX Pharmaceuticals, The Woodlands, TX). One month later, mice were sacrificed for immunogenicity studies or challenged with influenza (see below). Mice were housed and treated in a temperature-controlled, light-cycled facility at the University of Pennsylvania, and cared for under the guidelines of the NIH and the University of Pennsylvania.

#### Ferret Studies

Male ferrets (Triple F Farms, Sayre, PA), 4–6 month old, weight average 1.2±0.2 kg at the start of the study were immunized three times in alternate biceps femoris muscles, each one month apart, with 200 µg of DNA per antigen (and/or vector control) and electroporated as previously described [16]. Briefly, electroporation was delivered through a pentagonal 5-electrode array consisting of 21-gauge solid stainless steel electrodes. Three constant-current pulses of 0.5 Amps were delivered for 52 msec/pulse separated by a 1 sec delay using the CELLECTRA® adaptive constant current device (VGX Pharmaceuticals, The Woodlands, TX). Ferrets were housed and cared for at BIOQUAL, Inc. (Rockville, MD). These facilities are accredited by the American Association for the Accreditation of Laboratory Animal Care International and meet NIH standards as set forth in the Guidelines for Care and Use of Laboratory Animals.

#### Macaque Studies

Rhesus macaques were housed at BIOQUAL, Inc. (Rockville, MD), in accordance with the standards of the American Association for Accreditation of Laboratory Animal Care. Animals were allowed to acclimate for at least 30 days in quarantine prior to any immunization. A group of five rhesus macaques were immunized at weeks 0 and 4 with 1 mg/construct of pH5HA, pNP, and pN1NA. DNA was delivered into the semimebranosous muscle followed by *in vivo* electroporation using the constant current CELLECTRA® device (VGX Pharmaceuticals, The Woodlands, TX). Electroporation conditions were 0.5 Amps, 3 pulses, 52 msec pulse length, with 1 sec between pulses.

#### Blood collection

*Non-human primates*. Animals were bled every two weeks. 10 mL of blood was collected in EDTA tubes, and peripheral blood mononuclear cells (PBMC) isolated by standard Ficoll-hypaque centrifugation and re-suspension in complete culture medium (RPMI 1640 with 2 mM/L L-glutamine, 10% heat-inactivated fetal bovine serum, 100 IU/mL penicillin, 100 µg/mL streptomycin, and 55 µM/L β-mercaptoethanol). Red blood cells (RBC) were lysed with ammonium chloride-potassium (ACK) lysis buffer (Cambrex BioScience, East Rutherford, NJ).

#### ELISpot assay

ELISpot assays were conducted as previously described [17]. Briefly, ELISpot 96-well plates (Millipore, Billerica, MA) were coated with **a**) anti-mouse (R&D Systems, Minneapolis, MN) or **b**) anti-human (clone GZ-4, Mabtech, Cincinnati, OH) interferon- gamma (IFN-γ) capture antibody and incubated overnight at 4°C. The following day, plates were washed with PBS and blocked for 2 h with R10. 2×10^5^ splenocytes (mouse) or PBMCs (macaque) from each group were added to each well and stimulated overnight at 37°C in the presence of R10 peptide (negative control) or specific peptide antigens (10 µg/mL) (Invitrogen, Carlsbad, CA). Peptide pools consist of 15-mer peptides overlapping by 11 amino acids. After 24 h of stimulation, the cells were washed and incubated for 24 h at 4°C with **a**) anti-mouse IFN-γ Ab (R&D Systems, Minneapolis, MN) or **b**) anti-human (clone 7-B6-1, Mabtech IFN-γ capture antibody). Plates were washed, and streptavidin-alkaline phosphatase (R&D Systems, Minneapolis, MN) was added to each well and incubated for 2 h at room temperature. Plates were washed, and BCIP/NBT chromogen (R&D Systems, Minneapolis, MN) was added. The plate was then rinsed with distilled water and dried. Spots were counted by an automated ELISpot reader (CTL Limited, Shaker Heights, OH).

#### Antibody ELISA assay

96-well high-binding polystyrene plates (Corning, Lowell, MA) plates were coated overnight at 4°C with recombinant protein (2 µg/mL, XpressBio, Thurmont, MD) diluted in PBS. The next day, plates were washed with PBS, 0.05% Tween 20 (PBST), blocked for 1 h with 3% BSA/PBST, and incubated with serial dilutions of serum from immunized and naïve mice for 1 h at 37°C. Bound IgG was detected using goat anti-mouse IgG-HRP (Research Diagnostics, Concord, MA) at a dilution of 1∶10000. Bound enzyme was detected by the addition of the chromogen substrate solution TMB (R&D Systems), and read at 450 nm on a Biotek (Winooski, VT) EL312e reader.

#### Hemagglutination Inhibition (HI) Assay

Sera were treated with receptor-destroying enzyme (RDE) by diluting one part serum with three parts enzyme and incubated overnight in 37°C water bath. The enzyme was inactivated by 30 min incubation at 56°C followed by addition of six parts PBS for a final dilution of 1/10. HI assays were performed in V-bottom 96-well microtiter plates, using four hemagglutinating units (HAU) of virus and 1% horse RBC as previously described [18]. The viruses used for the HI assay are reassortant strains obtained from the Center for Disease Control and Prevention (CDC), Influenza branch (Atlanta, GA): A/Viet/1203/2004(H5N1)/PR8-IBCDC-RG and A/Indo/05/2005 (H5N1)/PR8-IBCDC-RG2.

#### Determination of Viral Titers for the ferret challenge study

For RNA isolation, nasal wash samples were spun down at 10,000×g for 1 hr, liquid poured off and 1 mL of RNA-STAT 60 (Isotex Diagnostics, Friendswood, TX) added. Samples were then incubated at RT for 5 min and resuspended in 250 µL of chloroform by vortexing. The samples were spun down at 10,000 g for 1 hr, the aqueous top-layer removed, 0.5 mL isopropanol and 10 µl tRNA (10 µg/mL) added and precipitated overnight at −20°C. Samples were spun down for 1 hr, washed with cold 75% ethanol and respun for another hour. RNA was resuspended in 30 µl RNAse-free water. For RT-PCR, 10% RNA was added to TaqMan reagents (Applied Biosystems, Foster City, CA) along with primers and probe (listed below) and amplified in a 7700 Sequence Detection System (Applied Biosystems). Briefly, the sample was reverse-transcribed at 48°C for 30 min., held at 95°C for 10 min., then run for 40 cycles of 95°C for 15 s and 60°C for 1 min. The signal was compared to a standard curve of known concentrations of RNA starting at 10^6^ down to 1 copy/mL and multiplied by 10 giving a detection range from 20–10^7^ copies/mL. All samples were performed in triplicate. The primers and probe were designed to bind to a highly conserved region on the nucleoprotein gene.

Primer sequences:

VIETA-U 5′ CGT CTC AAG GCA CCA AAC G 3′

VIETA-D 5′ GTA GAA CCT CCC AAT GCC AC 3′

Probe sequence: VIETA-P FAM-GGA ACG CCA GAA TGC TAC TGA GAT CAG GGC-TAMRA.

#### Virus Challenge

For the initial murine H5N1 influenza challenge, isoflurane-anaesthetized mice were intranasally inoculated with 100 LD_50_ of A/Hanoi/30408/2005 (obtained from CDC) in 50 µl MEM/3% BSA at the Public Health Agency of Canada. The murine H5N1 challenge analyzing correlates of immunity was conducted at BIOQUAL, Inc. using 10 LD_50_ of A/Vietnam/1203/2004. Murine H1N1 challenges were performed at the University of Pennsylvania. Avertin-anaesthetized mice were intranasally inoculated with 10 LD_50_ of A/Puerto Rico/8/34 in 30 µl PBS. CD4+ and CD8+ T cell depletions were performed using 150 µg of monoclonal antibodies GK1.5 (BD Pharmingen, San Jose, CA) or 2.43 (purified). All murine challenge groups were comprised of 10 mice per group. Ferret H5N1 challenges were performed at BIOQUAL, Inc., with 10^4.7^ EID_50_ of A/Vietnam/1203/2004. There were four ferrets in each group, and all ferrets tested seronegative for recently circulating H1N1 and H3N2 viruses. Both mice and ferrets were weighted daily. After challenge, weights, body temperature, and clinical signs were recorded daily from day −1 before challenge to day 9 after challenge and then on days 11 and 14 after challenge.

## Results

### Murine Immunogenicity and Challenge Studies

**Figure 1:**
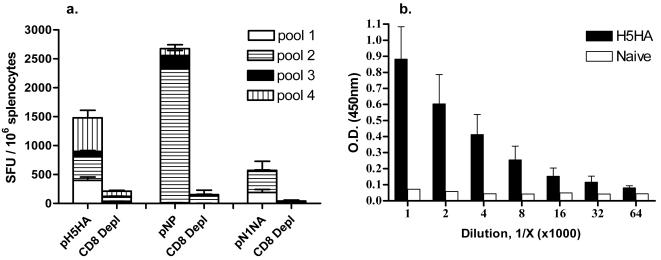
Induction of antigen-specific immune responses in BALB/C mice. (a) Quantification of IFN-γ secreting CD8+ T cells at one month following the final immunization. Splenocytes were stimulated with pools of overlapping peptides spanning the length of the antigen, separated into multiple pools. Also included are CD8-depleted controls. (b) Endpoint antibody ELISA from serum collected from mice immunized with pH5HA. All error bars represent ±1 standard deviation from the mean, and are representative of three independent experiments.

IVE appears to be a promising new technology for the delivery of DNA vaccines [1], [19], [20], [21], [22], [23], [24]. In order to determine if, combined with our consensus antigens, IVE could induce a potent immune response against synthetic DNA antigens, we immunized and electroporated BALB/C mice with each of the individual vaccine candidates. Figure 1a shows the ELISpot results from immunized mice. Both pH5HA and pNP are capable of inducing dramatic (interferon-γ-based) cellular immune responses, largely dependent upon CD8+ T cells, as shown by the loss of interferon-γ secretion in CD8-depletion controls, ELISpots for H5HA were 1478±209 vs. 209±39 in CD8-depleted mice (average ±SE mean), while ELISpots for pNP were 2676±145 vs. 151±201, in CD8-depleted mice. pN1NA was also able to elicit a strong cellular response, eliciting a mean of 571 spots ±384 vs. 41±40 in CD8-depleted mice. Splenocytes from naïve mice stimulated with these antigens, and R10 controls for, were all well below the 40-spot assay threshold (data not shown). In addition, Figure 1b shows the ability of pH5HA to induce a strong antibody response with an endpoint titer above 1∶64,000. In contrast, neither pN1NA nor pNP elicited any detectable antibody responses as determined by ELISA and HI assays (data not shown).

**Figure 2:**
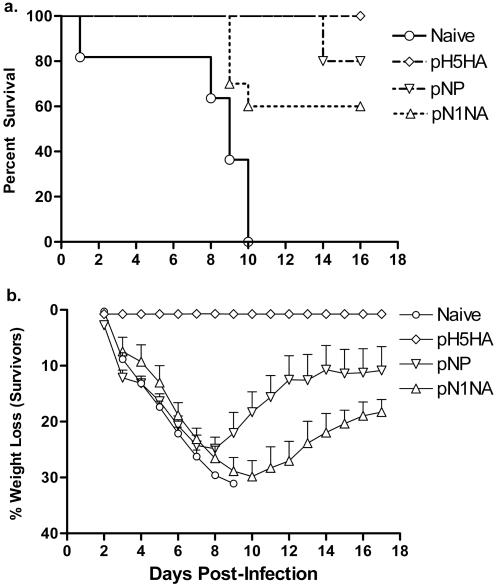
Results following an H5N1 challenge of immunized mice. Mice were challenged with 100LD_50_ of an H5N1 virus (A/Hanoi/05/2005). (a) Kaplan-Meier curve showing the percent survival in each group. (b) Average weight loss among the surviving members of each group (and the weight loss seen in naïve mice before death). Error bars represent 1 standard deviation from the mean, and are shown only in the positive direction for clarity.

In order to determine if the immune responses elicited by these synthetic antigens are capable of providing protection from HPAI, we challenged BALB/C mice with 100 LD_50_ of the highly pathogenic H5N1 virus A/Hanoi/30408/2005 (not included as a component sequence in the design of any consensus immunogen). Figure 2a shows the survival curve for each group of mice. pN1NA showed 60% protection from death, while pNP was able to protect from death 80% of the vaccinated animals, which also exhibited improved morbidity (decreased weight loss post-challenge, with the average maximum weight loss in the pNP group at 24.9%±2.1%, and in the pN1NA group at 29.9%±2.8%, as shown in Figure 2b). As shown, vaccination with pH5HA offered complete protection from both morbidity (with no significant weight loss detected) and mortality.

**Figure 3:**
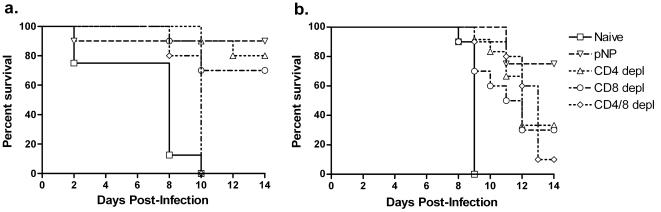
Kaplan-Meier survival curve in mice challenged with (a) H1N1 influenza (A/PR/8/34) and (b) H5N1 influenza (A/Vietnam/1203/04). All mice were immunized with pNP (except for naïve) and depleted of CD4+ T cells, CD8+ T cells, both, or neither.

We next decided to extend these findings to another murine haplotype while asking several questions regarding the observed protection. Previous murine studies of vaccination and challenge with influenza have shown that cellular immune responses can provide protection from infection-associated morbidity and mortality [25], [26], [27], [28]. It has also been shown that this protection can be mediated by either CD4+ or CD8+ T cells [25], [26]. This issue has not been addressed, however, in the context of HPAI, where severe cytokine dysregulation could potentially impact the function of effector T cell subset. In this second set of studies, we immunized and electroporated C57BL/6 mice with the pNP construct and challenged them after depletion of either CD4+ T cells, CD8+ T cells, both, or neither. These mice were challenged with an H1N1 virus (A/PR/8/34). As shown in Figure 3a, protection was seen individually with either CD4+ or CD8+ T cells, demonstrating *in vivo* that the synthetic vaccines can induce cross-reactive CD4+ and CD8+ cellular immune responses (90% survival in vaccinated, undepleted mice, 80% survival in CD4-depleted mice, 70% survival in CD8-depleted mice, and 0% survival in dual-depleted and naïve controls). Furthermore, challenging a separate group of mice with an H5N1 virus (Figure 3b) demonstrated that, in the context of pathogenic influenza and the associated cytokine dysregulation, both CD4+ and CD8+ subsets together provide considerably more protection than either alone (75% survival in vaccinated, undepleted mice, 36% survival in CD4-depleted mice, 38% survival in CD8-depleted mice, 11% survival in dual-depleted mice, and 0% survival in naïve mice). The slower onset of mortality in H5N1-infected, T cell-depleted mice may suggest that cellular immunity may play both a role in protection and pathogenesis of avian influenza.

### Induction of Cross-Reactive Antibodies

**Figure 4:**
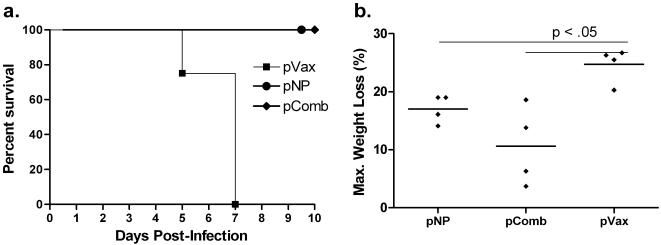
Survival and weight loss following H5N1 ferret challenge. (a) Kaplan-Meier curve of ferrets challenged with an H5N1 virus (A/Vietnam/1203/04). Ferrets were immunized and electroporated three times with the indicated constructs. One month following the final immunization, ferrets were challenged intranasally with a lethal dose of an H5N1 influenza virus. (b) Maximum weight loss in each group. Error bars represent 1 standard deviation from the mean, and are shown only in the positive direction for clarity.

**Figure 5:**
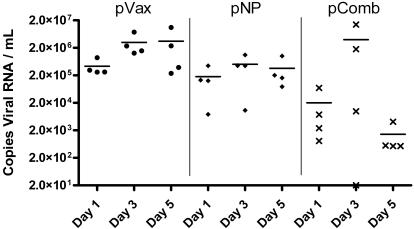
Viral shedding following challenge with HPAI. Nasal washes were collected every other day immediately following challenge. RT-PCR was performed to determine the RNA copy number from each group.

The ferret model of influenza infection is considered to be more reflective of human disease and a more rigorous challenge model. Ferrets exhibit similar symptoms to humans infected with influenza and similar tissue tropism with regards to human and avian influenza viruses [29]. In this study, three groups of ferrets were immunized and electroporated with 1) pVax (control), 2) a combination of pH5HA, pN1NA, and pNP (denoted as pComb in Figures 4 and 5), or 3) pNP alone. Serum was collected at different time points throughout the study to detect hemagglutination inhibition (HI) activity against H5N1 viruses, with HI titers of greater than 1∶40 considered reflective of a protective humoral immune response. As shown in Table 1, ferrets that received the pH5HA construct attained protective levels of antibody (mean of 1∶170, range 1∶40–320) after two immunizations. Following the third immunization, these titers increased to a mean of 1∶600 (range 1∶160–1280). We then tested the ability of these constructs to induce antibodies capable of inhibiting a more divergent H5N1 clade 2 virus. Again, after the third immunization, protective titers of HI activity reached 1∶210 (mean, range 1∶40–320). Importantly, we observed an HAI titer of greater than 1∶40 for both clade 1 and 2 viruses in all the animals receiving the consensus HA immunogen. These data demonstrate for the first time that synthetic DNA antigens can efficiently induce cross-reactive antibodies against H5N1 influenza in the ferret model.

### Ferret Challenge

One month following the third and final immunization, ferrets were challenged intranasally with a lethal dose of the HPAI virus A/Vietnam/1203/04. As shown in Figure 4a, all naïve animals had succumbed to infection by day 7, while all of the vaccinated animals in each group survived through the end of the study. Figures 4b shows weight loss in each of the infected groups. The highest degree of protection from weight loss was seen in those animals with both protective antibodies and broad cellular immune responses, losing 10.6% body weight ±3.4% (compared to 24.7%±1.5% in the control group, p<.05). pNP, which induces only potent cellular immune responses, was able to provide a statistically significant degree of protection, with an average weight loss of 17%±1.2% (p<.05). Figure 5 shows viral shedding from each group on days 1, 3, and 5 post-infection. Each vaccinated group displayed a statistically significant reduction in viral shedding by day 5 (p<.01). In group pComb, the mean viral load was reduced by over 99%. In the group pNP, with protection based solely on cellular immunity, there was a 90% reduction in mean viral load. These data support that the vaccine-induced cellular and humoral immune responses can impact disease and transmission (viral shedding) associated with pathogenic influenza infection in the more relevant ferret model.

### Primate Immunogenicity

**Figure 6:**
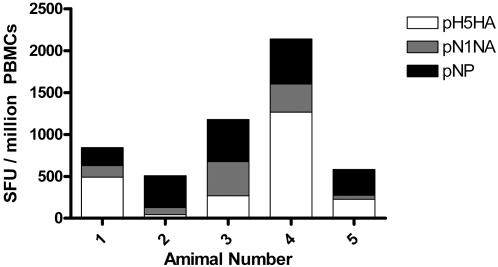
Induction of antigen-specific immune responses in rhesus macaques. Purified PBMCs were analyzed two weeks following the second immunization for antigen-specific interferon-γ responses. Values from media-stimulated controls were subtracted from the corresponding antigen-stimulated monkey samples.

Lastly, while these data provide important insights into the development of novel vaccine platforms appropriate for HPAI, the ability to induce these immune responses in larger animals will dictate their ability to move into clinical evaluation. We next studied the immunization and electroporation of rhesus macaques with the consensus influenza antigens. Figure 6 shows the ELISpot results after two immunizations. Each of the macaques generated strong responses against all three antigens, with a minimum ELISpot count of over 500 and a maximum above 2100 (mean spot count 1050±662 SEM).

**Figure 7:**
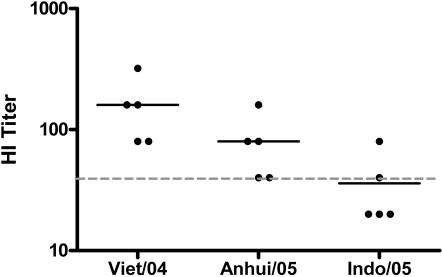
Hemagglutination inhibition activity in rhesus macaques following two immunizations. HI activity is shown for clade 1 (A/Vietnam/1203/04), clade 2.1 (A/Anhui/01/05), and a more divergent clade 2.1 (A/Indonesia/05/05). Naïve monkeys showed no detectable HI activity against any of the indicated strains.

Looking at hemagglutination inhibition activity following only two immunizations (Figure 7), we see that each of the monkeys demonstrated HI activity against a similar clade 1 virus, with mean titers of 1∶160 (range 1∶8–320). There is also significant inhibition of a clade 2.1 virus (A/Anhui/01/05, mean inhibition 1∶80, range 1∶40–160), and detectable inhibition in each macaque against a more divergent clade 2.1 virus (A/Indonesia/05/05, mean inhibition 1∶36, range 1∶20–80). The ability of DNA to induce potent and cross-reactive immune responses against influenza in primates is of considerable interest and may provide a useful tool to influenza prevention efforts.

## Discussion

The ability to induce cross-reactive cellular and humoral immune responses in humans would be a significant advance in the development of next-generation vaccines [30]. This is especially true for seasonal and pathogenic influenza–particularly H5N1 influenza viruses, which have become endemic in many countries. Consensus vaccines offer a novel means of inducing such responses. These vaccines are designed using a large number of primary viral sequences, and as such contain the most highly conserved characteristics of each. This is reflected in Table 2, which quantifies the advantages of using consensus constructs, and their increasing homology against primary strains of influenza. These constructs have been shown not only to induce strong CD8+ and CD4+ cellular immune responses, but that these responses are in fact more cross-reactive than their individual component antigens [31], [32], [33], [34]. However, a majority of these studies have focused on HIV, and can not realistically address the ability of these synthetic vaccines to induce protective antibody responses *in vivo*.

**Table 2:** Quantitative Comparison of Homologies Between Component and Consensus Sequences.

	NP	N1NA	H5HA
Minimum homology between any two component viruses	89	77.4	87
Minimum homology between component viruses and consensus	91.4	79.6	95.4
Average homology between component viruses and consensus	96.8	89	98.8

Quantitative description of consensus immunogen design. This table describes the advantages of using a consensus immunogen to protect against an unknown emerging influenza virus.

The absence of any prior immunity to H5N1 viruses significantly inhibited the initial development of subunit and kill-virus vaccines capable of demonstrating *in vitro* antibody reactivity to H5N1 viruses. Unfortunately, the continued evolution of H5N1 viruses increases the possibility that these stockpiled vaccines will have poor cross-reactivity to a virus eventually capable of crossing the species barrier. Because currently approved vaccination strategies require a significant production timeline, and given the mortality rate and emerging data on surveillance of H5N1 influenza evolution, significant measures need to be taken in anticipation of potential pandemics. DNA vaccines offer such an alternative, as they can be designed and produced more quickly than other available technologies.

Our results here demonstrate the ability of synthetic consensus constructs, with *in vivo* electroporation, to induce protective cellular and humoral immune responses in small and large animals models of vaccination. Studies with pH5HA in the mouse demonstrate the ability of synthetic consensus HA to provide immunity from a clade-matched pathogenic challenge. These responses, in both ferrets and macaques, also demonstrate the ability of synthetic consensus antigens to induce responses capable of inhibiting divergent viruses of the H5N1 subtype.

While antibody-based vaccines are the only current means by which to provide sterilizing immunity, a vaccine to reduce the morbidity and mortality associated with a rapidly spreading pathogenic virus would be a valuable tool in combating a pandemic.

In addition, reducing the viral load and duration of disease could also reduce the rate of viral transmission, giving public health officials additional time in a dangerous situation. And while simple point mutations can render an antibody-based vaccine ineffective, cellular immune responses, considerably more promiscuous than their antibody counterpart, and can recognize several variants of the same epitope.

In studying the pNP-induced correlates of immunity to H1N1 influenza in the mouse, we have shown here that these synthetic consensus vaccines are able to induce both CD4+ and CD8+ cellular immune responses capable of providing protection from influenza infection. Extending these experiments to an H5N1 murine challenge model suggested that these responses, while protective, are both required in responding to more highly pathogenic influenza viruses. Importantly, this cellular protection was reproducible in the more prognostic ferret model of pathogenic influenza infection, demonstrating for the first time significant protection from weight loss and viral shedding.

The ability of DNA to induce protective correlates of immunity to pathogenic influenza in a primate is an important step forward for this technology. These responses, induced after only two immunizations, can likely be enhanced with the addition of genetic adjuvants such as IL-12 and IL-15 that ours and other labs have reported on. These questions merit further study, as the ability to induce such responses with a single vaccination would be very useful in any vaccination campaign. The ability of these antigens to protect from both morbidity and mortality in a ferret model of HPAI, in both the presence and absence of neutralizing antibody, may present an advantage for a vaccine that seeks to overcome the antigenic drift that will likely occur before pandemic viruses cross the species barrier to humans.
